# *Call for Papers:* Health Equity

**DOI:** 10.1089/heq.2021.29011.cfp

**Published:** 2021-11-12

**Authors:** Xinzhi Zhang

**Affiliations:** National Heart, Lung, and Blood Institute, National Institutes of Health Bethesda, MD, USA


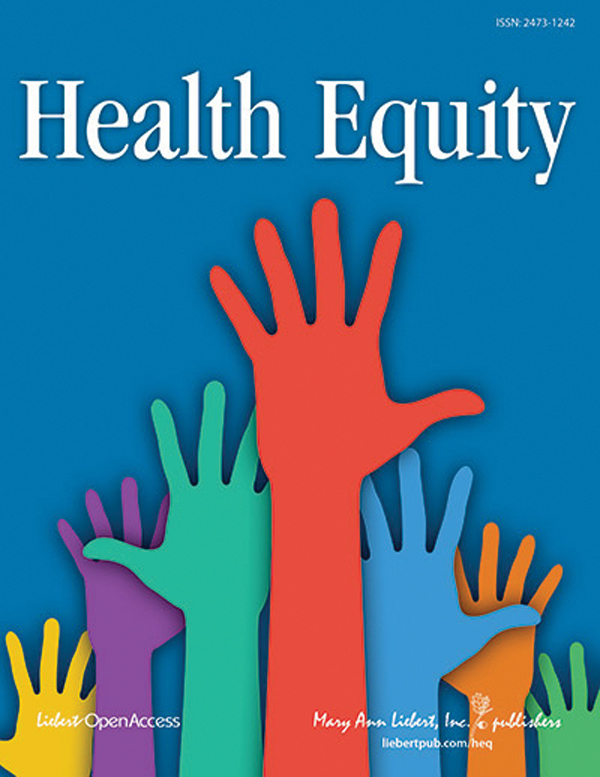


*Health Equity* invites you to submit your manuscript for publication today!

Benefits of publishing with *Health Equity* include:

- High visibility, immediate and unrestricted online access to published articles
- Rigorous and rapid peer review
- Easy compliance with open access mandates
- Authors retain copyright
- Citation tracking and inclusion in bibliographic databases — including Web of Science Emerging Sources Citation Index, Scopus, PubMed Central, among others
- Targeted email marketing
- 5% of all open access Article Processing Charges (APCs) are donated to the charity of your choice from the following five charities: UNICEF, National Medical Association, The Humane Society International, Nature Conservancy, and Doctors without Borders.

All articles in the Journal are rapidly reviewed and published online within 4 weeks of acceptance, and publish under the Creative Commons Attribution 4.0 (CC BY) license to ensure broad dissemination and participation.

Please visit our website at www.liebertpub.com/heq to view our Information for Authors.

Editorial or technical questions?

Contact Taylor Bowen (heq_eo@liebertpub.com)

**Visit the Instructions for Authors:**

www.liebertpub.com/heq

**Submit your paper for peer review online:**

https://mc.manuscriptcentral.com/heq

